# Vitamin D status and inflammatory Interleukins in women with and without polyendocrine metabolic ovarian syndrome: a cross-sectional exploratory study

**DOI:** 10.1080/07853890.2026.2749018

**Published:** 2026-10-14

**Authors:** Roba Bdeir, Nour Al-Sawalha, Hasan Rawashdeh, Ruba Malkawi, Ola Alrousan

**Affiliations:** a Department of Basic Scientific Sciences, Al-Balqa Applied University, Irbid, Jordan; b Department of Clinical Pharmacy, Faculty of Pharmacy, Jordan University of Science and Technology, Irbid, Jordan; c Department of Obstetrics and Gynecology, Faculty of Medicine, Jordan University of Science and Technology, Irbid, Jordan; d School of Pharmacy, Jadara University, Irbid, Jordan; e Department of Obstetrics and Gynecology, Jordanian Royal Medical Services, Prince Rashid Ben AlHasan Military Hospital, Aydoun, Jordan

**Keywords:** IL-6, IL-8, inflammation, Interleukins, polyendocrine metabolic ovarian syndrome, polycystic ovarian syndrome, vitamin D

## Abstract

**Background:**

Polyendocrine Metabolic Ovarian Syndrome (PMOS), formerly referred to as polycystic ovary syndrome (PCOS), is an intricate endocrine disorder, defined by a constellation of hormonal, metabolic, and reproductive irregularities. Vitamin D deficiency and increased levels of interleukin-6 (IL-6) and interleukin-8 (IL-8) have been implicated in the pathogenesis of PMOS. Although vitamin D deficiency and low-grade inflammation have each been associated with PMOS, the relationship between vitamin D status and circulating inflammatory cytokines remains incompletely characterized. Thus, this study aimed to investigate the association of Vitamin D and inflammatory interleukins believed to play a key role in the pathogenesis of PMOS.

**Method:**

The study involved 94 participants divided into two groups: 44 PMOS patients and 50 healthy controls. Age and BMI were recorded for each patient and plasma levels of Vitamin D, anti-Müllerian hormone (AMH), IL-6, and IL-8 were measured and compared between the two groups using the Enzyme-Linked Immunosorbent Assay.

**Results:**

AMH (*p* < 0.02), IL-6 (*p* < 0.001) and IL-8 (*p* < 0.001) levels were significantly higher in PMOS patients than in controls. Lastly, vitamin D levels were found significantly lower in PMOS patients (18.59 ng/mL) than in controls (37.95 ng/mL; *p* < 0.001). However, no significant correlation was found between IL-6, IL-8 and Vitamin D.

**Conclusion:**

Women with PMOS characterized by lower vitamin D concentrations alongside higher AMH, IL-6, IL-8, and BMI compared to healthy controls. Larger longitudinal and prospective interventional studies are required to clarify causality and determine if vitamin D supplementation improves clinical outcomes.

## Introduction

Polyendocrine metabolic ovarian syndrome (PMOS; formerly polycystic ovary syndrome [PCOS] [1],) is a complex endocrine condition that affects women of all ages and causes a range of symptoms related to hormone imbalance, including hyperandrogenism and ovarian dysfunction. Women throughout their reproductive years often experience it [2,3]. According to a recent thorough systematic review and meta-analysis, PMOS affects one in eight women worldwide during reproductive age [1,4]. Its clinical importance extends beyond reproductive dysfunction, as affected women have increased risks of metabolic abnormalities and long-term cardiometabolic complications. Numerous metabolic conditions, such as obesity and insulin resistance, are associated with PMOS. It has also been associated with chronic low-grade inflammation, which may contribute to the risk of long-term health issues like type 2 diabetes, cardiovascular disease, and certain types of cancer [5–8]. The magnitude of individual risks varies substantially according to age, BMI, insulin resistance, PMOS phenotype, and other comorbidities. This heterogeneity is important when interpreting circulating inflammatory and vitamin D biomarkers because metabolic factors such as BMI and insulin resistance may themselves influence inflammatory status and vitamin D concentrations.

Although PMOS is very common, its underlying causes remain incompletely understood. Insulin resistance and androgen excess are implicated in its pathophysiology by a substantial body of research. Numerous studies have shown that hyperandrogenism in PMOS patients is positively correlated with persistent low-grade inflammation [9,10]. In PMOS, follicular maturation typically stalls at the small antral stage, preventing ovulation and complete maturation. Hyperandrogenism, insulin resistance, and dysregulation of the hypothalamic-pituitary-ovarian axis such as luteinizing hormone (LH) > follicle-stimulating hormone (FSH) [11]. However, the mechanisms underlying these associations remain unclear.,

Patients with PMOS frequently have vitamin D deficiency, which can exacerbate many of the syndrome’s metabolic and reproductive symptoms including insulin resistance and hyperandrogenism, calcium dysregulation and follicular arrest, which is connected to menstrual irregularities and fertility issues [12]. Vitamin D regulates ovarian function, insulin sensitivity, and glucose metabolism—all of which are frequently compromised in PMOS. Serum levels of 25(OH)D are often lower in women with PMOS, according to studies [13,14]. Vitamin D can lower inflammatory responses and control the physiological balance by playing a critical role in maintaining the oxidative-antioxidant balance within the body [15,16].

Additionally, Vitamin D may play a critical role in decreasing inflammation and adjusting anti-Müllerian hormone (AMH) levels [17], which are typically greater in PMOS patients [18,19]. AMH is produced predominantly by granulosa cells of preantral and small antral ovarian follicles and is an important marker of ovarian follicular reserve. AMH limits excessive recruitment of primordial follicles and modulates follicular development. In PMOS, circulating AMH concentrations are frequently elevated, partly reflecting the increased number of small antral follicles characteristic of the ovarian phenotype. Elevated AMH may also contribute to disturbed follicular maturation and ovulatory dysfunction [20]. Thus, AMH may provide complementary information about the reproductive phenotype of PMOS in addition to metabolic and inflammatory biomarkers. Numerous animal studies have investigated the association between vitamin D supplements and PMOS. In particular, one study done on PMOS female rats revealed a drop in AMH serum levels when undergoing vitamin D treatment, demonstrating the essential role that vitamin D plays in the normal pattern of folliculogenesis [21]. Similarly, there were cases of serum AMH reduction in the study conducted by Irani and colleagues on PMOS-afflicted women undergoing vitamin D therapy. Conversely, blood anti-inflammatory soluble receptors for the byproducts of advanced glycation were more prevalent in PMOS patients with vitamin D deficiency [22].

In addition to hormonal and metabolic issues, women with PMOS have been found to have higher amounts of pro-inflammatory cytokines such as Interlukin-6 (IL-6), Interleukin −8 (IL-8) and tumor necrosis factor-alpha (TNF-α), in their serum and follicular fluid, which may be a contributing factor to the inflammatory ambience linked to the disorder [23–25]. The current associations between PMOS and immune cell alterations provide an explanation for the process of chronic low-grade inflammation. The most prevalent immune cells in ovaries and adipose tissues are macrophages, which balance protective and destructive cell-mediated immunity in inflammation [26], possibly linking obesity and/or insulin resistance with the chronic low grade inflammation symptoms of the disease.

Specifically, IL-8 possibly has an impact on ovarian function since it promotes follicular maturation and ovulation [27]. In a clinical trial, IL-8 expression rose at first, but it fell after pioglitazone and metformin were administered [25]. The granulosa cells’ IL-8 levels rose when BMI increased in contrast to obese PMOS and non-PMOS patients, however serum’s IL-8 levels fell. Granulosa cells are responsible for the secretion of AMH and plays an essential role in regulating a hormonal balance, because it inhibits the transition from primordial to primary follicles [28,29]. One mechanism of PMOS may entail disrupting the natural ovarian hormone synthesis, which leads to the hormonal imbalances characteristic of PMOS, by dysregulation of IL-8.

Looking more into the other pro-inflammatory cytokines, even though all cell types include the pleiotropic signaling inflammatory marker IL-6 [30], in the context of PMOS, it specifically regulates sex hormone synthesis, fetal development, and corpus luteum activity [31]. Serum IL-6 levels were shown to be elevated in PMOS patients with insulin resistance, while androgens have been shown to trigger immunological reactions in female PMOS patients with obesity. According to a study, IL-6 has negative consequences and is higher in infertile PMOS women than in healthy controls [32].

Nonetheless, there is still lack of clear understanding and limited number of studies exploring such inflammatory markers, especially IL-8, and their link to PMOS [33,34]. Although vitamin D deficiency and low-grade inflammation have each been associated with PMOS, the relationship between vitamin D status and circulating inflammatory cytokines remains incompletely characterized. In particular, relatively few studies have simultaneously evaluated vitamin D, IL-6, IL-8, and AMH within the same cohort, and evidence regarding the relationship between vitamin D and IL-8 is limited and inconsistent.

Thus, this study aimed to evaluate serum levels of AMH, vitamin D, IL-6 and IL-8 in women diagnosed with PMOS in comparison to healthy controls. Furthermore, it aimed to investigate the association of Vitamin D and inflammatory interleukins believed to play a key role in the pathogenesis of PMOS.

## Methods

### Sample size and statistical power

The prevalence of polycystic ovary syndrome (PMOS) varies, with estimates ranging from 6% to 13% of reproductive-aged women. Sample size was determined a priori for a cross-sectional comparison between women with PMOS and healthy controls. Assuming a two-sided significance level of 0.05, 80% statistical power, equal allocation between the two groups, and a moderate standardized effect size (Cohen’s *d* = 0.50), the estimated minimum sample size was 128 participants (64 participants per group). Due to the limited availability of eligible participants during the study period, a total of 94 participants were recruited, including 44 women with PMOS and 50 healthy controls. The smaller sample size may have reduced the statistical power to detect smaller effect sizes and associations, particularly in secondary correlation analyses.

### Participants and study design

The study was approved on May 8, 2025 by the Institutional Review Board from Al-Balqa Applied University with approval reference number: 103/8/2024/2025. Between June and August of 2025, a total of ninety-four participants were selected from a private Gynecology clinic in Irbid, Jordan, to participate in this cross-sectional study. Thus, all samples were collected within the summer season in Jordan; therefore, the findings reflect vitamin D status during this period and may not be directly generalizable to other seasons. A written informed consent was obtained from all subjects involved in the study. Participants were divided into two groups: 50 healthy volunteers made up the control group, and 44 women with a confirmed PMOS diagnosis made up the patients group. The assessment tool for PMOS diagnosis used was the Rotterdam criteria [35,36], for adult women it requires the presence of at least two of the three manifestations: polycystic ovarian appearance on ultrasound, clinical and/or biochemical hyperandrogenism, and oligomenorrhea. For every individual, their age and BMI were noted. Age was matched between the PMOS and control groups. The control group consisted of women without a previous diagnosis of PMOS who reported regular menstrual cycles and had both ovaries present. Women with a history of PMOS, confirmed infertility, overt autoimmune disease, chronic metabolic or endocrine disorders, or recent hormonal therapy were excluded. Participants using medications that could substantially alter hormonal or biochemical parameters, including metformin, pioglitazone, statins, or antihypertensive medications, were also excluded. PMOS.

### Biochemical investigations

Blood samples were collected between 10:00 and 15:00. Approximately 10 mL of peripheral venous blood was drawn from each participant and placed in a plain blood tube. Because collection was not restricted to a narrow time window, potential effects of diurnal variation cannot be completely excluded. Participants were instructed to fast overnight for 8–12 h before blood collection, with water permitted. The serum was then refrigerated at −20 °C after being separated using a cooling centrifuge set to 765 g and 4 °C for five minutes. Total 25-hydroxyvitamin D (25(OH)D) levels and anti-Mullerian hormone (AMH) were measured with the CL-900i Chemiluminescence Immunoassay System (Mindray, China). Finally, using a commercial Enzyme-Linked Immunosorbent Assay (ELISA) Kits supplied by Cloud-Clone Corp, total concentrations of IL-6 (Houston, USA, Cat. No. SEA079Ra) and IL-8 (Houston, USA, Cat. No. HEA080Hu) in serum samples were measured in accordance with the manufacturer’s instructions. Vitamin D-binding protein and albumin were not measured; therefore, the present study evaluated total circulating 25(OH)D rather than free or bioavailable 25(OH)D.

### Ethical considerations

The study protocol was approved on May 8, 2025 by the Institutional Review Board (IRB) with approval reference number: 103/8/2024/2025. Clinical trial number: not applicable. Informed consent was obtained from every participant. Participants’ personal information, including their name and phone number, were left out to ensure anonymity.

### Statistical analysis

Data were analyzed using SPSS version 26 (SPSS Inc., Chicago, IL, USA). The normality of the distribution was confirmed using the Kolmogorov-Smirnov test. Ranges of minimum and maximum, mean ± standard deviation were used to characterize quantitative data. The Mann-Whitney test or the Student’s t test, whenever appropriate, were used to examine the mean differences between the two groups. Spearman’s correlation test was employed to show whether there was a significant correlation between continuous and discrete variables. *P*-values were deemed statistically significant if they were less than 0.05.

## Results

### Patient clinical characteristics

A total of 44 patients with confirmed PMOS enrolled into the study, and 50 healthy women served as a control group. Participants ranged from 18 till 46 years. The details of the participants’ age and BMI are presented in Table 1. The patients included in this group did not receive any form of therapeutic intervention during the study period. This means they were not administered any medication, surgical procedures, physical therapy, or other clinical treatments intended to alter the course of their condition. A statistically significant elevation in BMI was observed in PMOS patients (29.5 ± 2.11 kg/m^2^) compared to healthy controls (27.1 ± 1.93 kg/m^2^; *p* < 0.001).

**Table 1.** Main clinical and inflammatory biomarkers in healthy control (*n* = 50) and PMOS groups (*n* = 44).

Variables	PMOS	Control	Test of Sig.	95% CI for differences	*p*-value
**Age (y)**	30.65 ± 5.55	29.44 ± 4.74	*t* = 1.531	−0.37 to 2.79	0.130
**BMI (kg/m^2^)**	29.5 ± 2.11 (25.0 − 32.8)	27.1 ± 1.93 (23.5 − 31.5)	*t* = 5.761	1.57 to 3.23	<0.001*
**AMH (ng/mL)**	5.96 (3.0 − 9.5)	2.53 (0.5 − 5.5)	*U* = 588.5*	1.67 to 5.19	0.020*
**IL-8 (pg/mL)**	25.15 (6.9 − 45.45)	6.19 (1.19 − 14.16)	*U* = 119.5*	10.11 to 27.81	<0.001*
**IL-6 (pg/mL)**	8.35 (2.05 − 15.95)	1.53 (0.50 − 5.54)	*U* = 195.0*	3.60 to 10.04	<0.001*
**Vitamin D (ng/mL)**	18.59 (6.74 − 35.8)	37.95 (20.4 − 52.4)	*U* = 256.0*	−28.51 to −10.21	<0.001*

Normally distributed variables are presented as mean ± standard deviation (SD), whereas non-normally distributed variables are presented as median (minimum–maximum). Between-group comparisons were performed using the independent-samples Student’s t-test or Mann–Whitney U test, as appropriate. The 95% CIs for Age and BMI represent the difference in means, whereas those for AMH, IL-8, IL-6, and Vitamin D represent the Hodges–Lehmann estimate of the median difference. A two-sided p-value <0.05 was considered statistically significant.

Furthermore, Table 1 presents the AMH, IL-6, IL-8 and vitamin D levels in the control and PMOS groups. AMH levels were significantly higher in PMOS patients (5.96 ng/mL) than in controls (2.53 ng/mL; *p* = 0.02). Both IL-8 and IL-6 levels were significantly higher in PMOS patients (25.15; 8.35) than in controls (6.19; 1.53, respectively) with a significant *p* < 0.001. Lastly, vitamin D levels were found significantly lower in PMOS patients (18.59) than in controls (37.95; *p* < 0.001)

### Correlation of clinical and inflammatory biomarkers between the two groups

The analysis of the correlation between IL-8 and Vitamin D levels in the patient group revealed a Spearman rank correlation coefficient (rs) of −0.255 with a *p*-value of 0.09, while between IL-6 and Vitamin D a Spearman rank correlation coefficient (rs) of −0.295 with a *p*-value of 0.106. This implies that Vitamin D has a weakly negative correlation with IL-8 and IL-6, meaning that lower levels of IL-8 and IL-6 may be linked to higher levels of vitamin D. The p-value, however, is higher than the standard cutoff point of 0.05, indicating that this link is not statistically significant. Consequently, while there is an indication of a potential inverse relationship, the evidence is insufficient to conclude a meaningful correlation between IL-8 and IL-6 with Vitamin D within this patient group. This could be due to the small sample size, which limits statistical power to detect modest associations, as well as variations among participants in lifestyle, PMOS severity, and other underlying factors. Table 2 shows the insignificant results for the Spearman rank correlation coefficient between IL-8, IL-6 and Vitamin D with different parameters.

**Table 2.** Correlation analysis of IL-6, IL-8, and Vitamin D with selected clinical parameters.

	IL-8 (pg/mL)		IL-6 (pg/mL)		Vitamin D (ng/mL)	
	r_s_*	*p*	r_s_	*p*	r_s_	*p*
**Age (years)**	−0.160	0.330	−0.159	0.434	0.239	0.144
**AMH**	0.077	0.643	0.055	0.535	0.315	0.051
**BMI (kg/m^2^)**	0.066	0.691	0.099	0.665	−0.129	0.435

*Spearman coefficient.

## Discussion

Vitamin D deficiency and chronic low-grade inflammation have been implicated in the pathophysiology of PMOS, particularly through their potential effects on insulin resistance and metabolic dysfunction [37,38]. Although studies have reported inconsistent associations between Vitamin D status and PMOS, differences in diagnostic criteria, geographic sun exposure, dietary patterns, and sample size may partly explain these findings [13]. Similarly, elevated pro-inflammatory cytokines, including IL-6 and IL-8, have been reported in women with PMOS, with some evidence suggesting that IL-6 is elevated independent of BMI, whereas IL-8 may vary according to BMI [39–42]. Our findings of increased IL-6 and IL-8 are consistent with previous reports linking Vitamin D deficiency and increased inflammatory markers in PMOS.

This study aimed to investigate some clinical and biochemical markers, including age, BMI, Vitamin D levels, AMH, IL-6 and IL-8, in women with PMOS compared to a control group. Also, it aimed to investigate the association of Vitamin D and inflammatory interleukins believed to play a key role in the pathogenesis of PMOS compared to a control group, revealing significant differences between these two groups in age, BMI as well as the levels of Vitamin D, AMH, IL-6 and IL-8. The mean age of PMOS patients in this study was 30.65 ± 5.55 years, while the control group averaged 29.44 ± 4.74 years. This finding is consistent with previous research indicating that PMOS often manifests during reproductive years, typically in women in their late twenties to early thirties [43].

The study also reported a statistically significant increase in BMI among PMOS patients (29.5 ± 2.11 kg/m^2^) compared with controls (27.1 ± 1.93 kg/m^2^), with a *p*-value of <0.001. This aligns with [44], who found that high BMI is prevalent in PMOS populations, with approximately 41% of their cohort classified as obese. The increased BMI in PMOS patients may be attributed to insulin resistance, which is a common feature of the syndrome and contributes to weight gain and difficulty in weight management [45].

Vitamin D levels were significantly lower in PMOS patients 18.59 ng/mL with a range between 6.74 − 35.8 ng/ml compared to controls 37.95 (20.4 − 52.4) ng/mL, with a *p*-value of < 0.001. This finding supports the work of Morgante and colleagues [13], who conducted a systematic review indicating that Vitamin D deficiency is common among women with PMOS and may contribute to metabolic dysfunctions associated with the syndrome. Vitamin D is thought to play a role in insulin sensitivity, and its deficiency may exacerbate insulin resistance, a hallmark of PMOS [46]. Conversely, some studies have reported no significant differences in Vitamin D levels among PMOS patients. For example, a study done in Rajasthan, India, found that while Vitamin D deficiency is prevalent in PMOS, its levels did not significantly differ from those in healthy controls [40]. One important consideration is that vegetarianism is the most common eating pattern among Indians, which may have an effect on vitamin D levels. This inconsistency may be attributed to factors such as geographic location, dietary habits, sun exposure, and the timing of blood sample collection, which can all influence Vitamin D status. Several methodological and population-related factors also contribute to discrepancies within literature, where the definitions and cutoff values used to classify vitamin D deficiency, variation in vitamin D assays and laboratory methods. In addition, differences in BMI distributions, dietary vitamin D intake, and the use of vitamin D supplementation may contribute to variability in circulating vitamin D concentrations. Differences in PMOS phenotypes and clinical characteristics, as well as variations in sample size and statistical adjustment strategies, may also influence the observed associations. Collectively, these factors should be considered when comparing vitamin D findings across studies.

More recent evidence further supports the potential relationship between vitamin D status and inflammatory activity in PMOS [47]. Wang et al. (2026), in a large prospective discovery cohort (580 women with PMOS, 332 controls), reported that lower serum 25-hydroxyvitamin D levels correlated more strongly with inflammatory markers, uterine perfusion indices, recurrent miscarriage, and live birth rates than HOMA-IR after controlling for confounding variables. While these findings strengthen the biological plausibility of an association between vitamin D status and inflammatory or reproductive features of PMOS, the present study did not demonstrate statistically significant correlations with IL-6 or IL-8. This variance my stem from differences in study design, cohort size, participant demographics, vitamin D distributions, inflammatory markers assessed, or statistical adjustment. Importantly, our findings should therefore be interpreted as evidence of group-level variations in vitamin D and inflammatory markers rather than a definitive causal link within the follicular microenvironment of women with elevated AMH levels.

Our study found elevated AMH levels in PMOS patients 5.96 ng/mL with a range between 3.0 − 9.5 ng/mL compared to controls 2.53 ng/mL (0.5 − 5.5), with a statistically significant *p*-value of < 0.02. This result is consistent with [48,49], who identified increased AMH as a characteristic feature of PMOS, reflecting an increased ovarian follicle count. Elevated AMH levels are often used as a diagnostic marker for PMOS, as they correlate with the number of antral follicles in the ovaries [50,51]. However, the relationship between AMH and BMI has shown inconsistent results in the literature. Specifically, two studies conducted in Indian and Chinese population [52,53] suggest that while AMH levels are elevated in PMOS, they may not be significantly influenced by BMI. This suggests BMI might differentially regulate AMH levels in lean and obese PMOS. This could indicate that AMH serves as a more specific marker of ovarian function rather than a direct reflection of metabolic status, suggesting that hormonal and metabolic aspects of PMOS may operate through different pathways.

Furthermore, our finding of elevated AMH concentrations in women with PMOS is also relevant to emerging evidence linking AMH with the ovarian oxidative environment [54]. Vale-Fernandes et al. reported higher circulating and follicular-fluid AMH concentrations in women with PMOS undergoing IVF and found that higher levels of circulating AMH were correlated with lower total antioxidant capacity in the follicular fluid, indicating that an environment with elevated AMH may be more vulnerable to oxidative stress [20,54]. Although oxidative-stress biomarkers were not measured in the present study, these findings provide a potential mechanistic context for the elevated AMH observed in our cohort and emphasize that AMH may reflect more than ovarian follicle number alone. Future studies integrating AMH, vitamin D, inflammatory cytokines, and direct measures of follicular or systemic oxidative stress may help clarify the relationships among these pathways.

Levels of IL-8 were significantly higher in PMOS patients 25.15 pg/mL with a range of 6.9 − 45.45 pg/mL compared to controls 6.19 pg/mL (1.19 − 14.16), with a p-value of <0.001. This supports the findings of Vasyukova and colleagues, which described the role of chronic inflammation in the pathophysiology of PMOS [33]. Elevated IL-8 levels may indicate an inflammatory state that contributes to insulin resistance and other metabolic disturbances commonly observed in PMOS patients. Interestingly, the current study found no correlation between IL-8 and Vitamin D levels in either group, with *p*-values of 0.077 in the patient group and 0.596 in the control group. There is a notable lack of literature specifically examining the relationship between IL-8 and vitamin D deficiency in women with PMOS. While numerous studies have explored the role of vitamin D supplements in modulating inflammation in PMOS, typically focusing on markers like C-reactive protein, TNF-α, and IL-6 [15,55], IL-8 is rarely assessed directly. In one of the few randomized controlled trials that included IL-8, vitamin D supplementation significantly reduced TNF-α and oxidative stress markers, but had no significant effect on IL-8 gene expression [56]. Overall, the available evidence does not establish a clear relationship. Because of this, there is currently insufficient data to draw a firm link between IL-8 and vitamin D levels in PMOS, underscoring the need for further focused studies.

Overall, this study reinforces the distinct clinical and biochemical profiles of PMOS patients compared to healthy controls. The significant differences in BMI, Vitamin D, AMH, IL-6 and IL-8 levels underscore the multifaceted nature of PMOS. However, the contradictions in literature regarding the relationships between these markers highlight the complexity of PMOS and the need for further research to elucidate their interactions and implications for diagnosis and treatment. One of the main limitations of our study is a single-center design and relatively small sample size, which may introduce selection bias not fully representing the general population and clinical settings, and limit the statistical power to detect subtle associations, particularly between vitamin D levels and specific inflammatory markers such as IL-8. Secondly, lifestyle factors that may influence both vitamin D status and inflammation, including dietary intake, sun exposure, physical activity, and potentially supplement use, were not comprehensively assessed. Finally, vitamin D and inflammatory cytokines were measured at a single time point, which limits assessment of intra-individual variability and temporal relationships. Because these biomarkers were measured in peripheral blood, they may not directly reflect the inflammatory or oxidative environment within ovarian or follicular tissues.

Future studies should use larger multicenter cohorts with longitudinal sampling to determine whether changes in vitamin D status precede changes in inflammatory biomarkers. Randomized controlled trials of vitamin D supplementation, ideally stratified according to baseline vitamin D status and BMI, could determine whether correction of deficiency modifies IL-6, IL-8, metabolic parameters, AMH, or reproductive outcomes. Integration of dietary intake, sun exposure, physical activity, insulin resistance, androgen status, and direct oxidative-stress biomarkers would further clarify the biological pathways linking vitamin D, inflammation, AMH, and PMOS.

## Conclusion

In this exploratory cross-sectional study, women with PMOS had significantly lower circulating vitamin D concentrations and higher AMH, IL-6, IL-8, and BMI than healthy controls. However, vitamin D concentrations were not significantly correlated with IL-6 or IL-8 within the PMOS group. These findings support the presence of distinct endocrine, metabolic, and inflammatory profiles in PMOS but do not establish a causal relationship between vitamin D status and inflammation. Given the potential influence of BMI, lifestyle, season, and other clinical factors, larger longitudinal studies with comprehensive adjustment for confounders are warranted. Prospective interventional studies are also needed to determine whether correction of vitamin D deficiency modifies inflammatory biomarkers, metabolic features, or reproductive outcomes in PMOS.

## Data Availability

The data that support the findings of this study are available from the corresponding author upon reasonable request. Due to ethical and privacy restrictions involving patient data, the datasets are not publicly available.
